# Supplementary material for: Cloud BioLinux: pre-configured and on-demand bioinformatics computing for the genomics community
Source: BMC Bioinformatics. 2012 Mar 19;13:42. doi: 10.1186/1471-2105-13-42 (PMC3372431; doi:10.1186/1471-2105-13-42)
Supplement: Additional file 1 — Supplementary 1 Cloud BioLinux software documentation in the form of a mini, self-contained website. Users need to download and uncompress the .zip file, and open through a web browser the "index.html" file available on the main directory. (ZIP 1823 kb). [file 1471-2105-13-42-S1.ZIP › Cloud-BioLinux-Package-Documentation/docs/consense.html]

Bio-Linux Software Documentation Pages

Back to search form

## consense

|  |  |
| --- | --- |
| Name | consense |
| Description | **consense** is part of the PHYLIP package  Copyright 1986-2004 by The University of Washington. Written by Joseph Felsenstein. Permission is granted to copy this document provided that no fee is charged for it and that this copyright notice is not removed.  **CONSENSE** reads a file of computer-readable trees and prints out (and may also write out onto a file) a consensus tree. At the moment it carries out a family of consensus tree methods called the Ml methods (Margush and McMorris, 1981). These include strict consensus and majority rule consensus. Basically the consensus tree consists of monophyletic groups that occur as often as possible in the data. If a group occurs in more than a fraction l of all the input trees it will definitely appear in the consensus tree.  The tree printed out has at each fork a number indicating how many times the group which consists of the species to the right of (descended from) the fork occurred. Thus if we read in 15 trees and find that a fork has the number 15, that group occurred in all of the trees. The strict consensus tree consists of all groups that occurred 100% of the time, the rest of the resolution being ignored. The tree printed out here includes groups down to 50%, and below it until the tree is fully resolved.  The majority rule consensus tree consists of all groups that occur more than 50% of the time. Any other percentage level between 50% and 100% can also be used, and that is why the program in effect carries out a family of methods. You have to decide on the percentage level, figure out for yourself what number of occurrences that would be (e.g. 15 in the above case for 100%), and resolutely ignore any group below that number. Do not use numbers at or below 50%, because some groups occurring (say) 35% of the time will not be shown on the tree. The collection of all groups that occur 35% or more of the time may include two groups that are mutually self contradictory and cannot appear in the same tree. In this program, as the default method I have included groups that occur less than 50% of the time, working downwards in their frequency of occurrence, as long as they continue to resolve the tree and do not contradict more frequent groups. In this respect the method is similar to the Nelson consensus method (Nelson, 1979) as explicated by Page (1989) although it is not identical to it.  The program can also carry out Strict consensus, Majority Rule consensus without the extension which adds groups until the tree is fully resolved, and other members of the Ml family, where the user supplied the fraction of times the group must appear in the input trees to be included in the consensus tree. For the moment the program cannot carry out any other consensus tree method, such as Adams consensus (Adams, 1972, 1986) or methods based on quadruples of species (Estabrook, McMorris, and Meacham, 1985).  **References:**  Felsenstein, J. 1993. PHYLIP (Phylogeny Inference Package) version 3.5c. Distributed by the author. Department of Genetics, University of Washington, Seattle.    Felsenstein, J. 1989. PHYLIP -- Phylogeny Inference Package (Version 3.2). Cladistics 5: 164-166. |
| Homepage | http://evolution.genetics.washington.edu/phylip.html |
| Remote Documentation | http://evolution.genetics.washington.edu/phylip/doc/consense.html |
